# Game Analysis on the Evolution of Decision-Making of Vaccine Manufacturing Enterprises under the Government Regulation Model

**DOI:** 10.3390/vaccines8020267

**Published:** 2020-05-31

**Authors:** Na Zhang, Yingjie Yang, Xiaodong Wang, Xinfeng Wang

**Affiliations:** 1 School of Economics and Management, Shihezi University, Shihezi 832000, China; zhangnanuaa@163.com; 2 Guanghua School of Management, Peking University, Beijing 100871, China; 3 Institute of Artificial Intelligence, De Montfort University, Leicester LE1 9BH, UK; yyang@dmu.ac.uk; 4 Business School, Zhengzhou University of Aeronautics, Zhengzhou 450046, China; 5 College of Animal Science and Technology, Shihezi University, Shihezi 832000, China; wxf-4@163.com

**Keywords:** government regulation, defective vaccines, severe punishment, evolutionary game

## Abstract

The harm caused by defective vaccines to human health and social stability is immeasurable. Aiming at the government’s supervision of the vaccine market, an evolutionary game model is constructed to analyze the quality of supervision and the key factors in the dynamic interaction between government departments and vaccine manufacturers under different supervision modes in the vaccine manufacturing process. The results show that: (1) Severe punishment by government regulatory authorities, and increased costs of rectification after investigation and handling of involuntary behaviors of vaccine enterprises can effectively prevent involuntary behaviors of vaccine enterprises. (2) In the early stage of the game, the success rate of the government’s efficient supervision will make the vaccine enterprises continuously self-disciplined; when the vaccine market is relatively stable, the government’s supervision departments tend to be more conducive to passive supervision. (3) The success rate of government regulatory departments and the probability of a third-party reporting to play a great role in promoting the self-discipline of enterprises. (4) The power of government and regulation are conducive to promoting the active supervision of the government regulatory authorities but corruption of government and awareness of people are different. Once the phenomenon of vaccine enterprises’ non-discipline increases, the government regulation must change from passive regulation to active regulation. Therefore, the government should implement different measures according to the characteristics of each period in the manufacturing process to effectively prevent problematic vaccines. The conclusions and policy recommendations are significant for addressing the issue of insufficient self-discipline of vaccine manufacturers.

## 1. Introduction

For more than two thousand years, human beings have been searching for ways to fight all kinds of infectious diseases. Vaccine immunization is an economical and effective way to curb infectious diseases, and many diseases have been effectively controlled by vaccine immunization [1]. However, in recent years, “defective vaccine” incidents have emerged one after another in Anhui province, Jiangsu province, Dalian city, Hebei Province, Shanxi province, Shandong province, Changchun city, Wuhan city, and other places in China, causing people to panic about the safety of vaccines [2,3,4], and resulting in an unprecedented trust crisis for vaccine enterprises. When the shadow of the milk powder incident and the food safety incident has not been completely removed, “defective vaccine” incidents will undoubtedly add insult to the injury. As the most economical and effectual medicines for the prevention and control of infectious diseases, vaccines are of great strategic significance for individual health, social public health, and social stability [5]. How to supervise vaccine enterprises more effectively has become the focus for all sectors of society [6]. Government regulation is the primary approach to solve vaccine safety problems [7].

In recent years, many experts and scholars have studied vaccine regulatory events, regulatory approaches, and regulatory models. Vaccine safety research is an important component of public health program [8,9,10,11]. Some scholars discussed the mutual restriction and interaction between the government and drug enterprises in the game process in order to find the reasons for the existence of unsafe drug incidents and put forward countermeasures [12]. Literature [13,14] discussed the game relationship between the government and production enterprises, local governments, and social supervision, and then put forward corresponding countermeasures from the aspects of strengthening supervision, clarifying the relationship between regulatory departments and local governments, and promoting the application of new technologies. Literature [15,16,17] argued that the government should not only adopt severe punishment for drug safety supervision, but also improve government supervision technology, advocate diversified regulatory bodies, and pay attention to enterprise self-control and industry self-discipline. Literature [18], using the analytical method of regulation theory, believed that drug regulatory authorities should strengthen supervision of drug production process, increase punishment for violations, reduce the cost of quality regulation, and improve the accountability for malfeasance and irresponsibility. Some scholars mainly studied regulatory agencies, and found that the media and the public supervision were an effective complement to the government regulation. Literature [19,20,21,22] put forward that strengthening media supervision and penalties can raise the level of drug safety in China by analyzing the game relationship between the governments, drug regulatory department, and the social public.

At present, scholars’ research on government regulation and vaccine quality and safety supervision is still insufficient. Firstly, with the development of society and the continuous awakening and improvement of public safety awareness, it is imperative for third-party regulatory agencies to participate in vaccine quality and safety supervision. The studies on third-party regulatory agencies participating in vaccine quality and safety have not received enough attention so far. Secondly, the government’s supervision of vaccine manufacturers pays more attention to the difference between active supervision and passive supervision instead of that between supervision and non-supervision before. Thirdly, in the existing game models, the government supervision department is regarded as a rational economic man who pursues the maximization of his own interests. This assumption is not consistent with the actual situation. In reality, the government supervision department aims at minimizing social losses. Therefore, under the assumption of bounded rationality of government supervisory authorities and vaccine manufacturers, this paper will analyze the equilibrium results by applying evolutionary game theory to vaccine quality and safety supervision. It is supposed to make the enterprise as self-disciplined as possible through the joint efforts of the government and the third party, and provides the basis for the government to formulate and improve various prevention policies and measures.

There is a famous “broken window effect” in criminal psychology. If someone breaks the window glass of a building and the window is not repaired in a timely manner, others may be encouraged by some demonstration to break more windows [23,24,25,26]. Over time, these broken windows create a sense of disorder, and crime flourishes in an atmosphere of public indifference. “Defective vaccines” of vaccine manufacturers are like cracked windows. Once one enterprise manufactures “defective vaccines” based on its own interests, if the government or the public fails to supervise and find out, other enterprises will follow suit and eventually cause “defective vaccines” to flourish. Vaccine regulation is a typical non-cooperative game problem. As an autonomous economic entity, the choice of vaccine enterprises seems rational on the surface, but it may not be reasonable from the perspective of the whole society. The addition of external intervention mechanism will effectively promote the system to achieve the optimal solution. Therefore, participation, coordination, and guidance of the government and the public are essential in solving the problem of vaccine regulation. Evolutionary game theory is a method that combines game and dynamic evolution, which can study the stable structure of the game system and the process of strategy selection of behavior subjects in the evolutionary process by introducing dynamic mechanism [27,28,29,30]. The basic idea is the case that in a group of a certain size, game players are not super rational players, and it is difficult to find the optimal equilibrium point in a single game, but multiple execution of the game can identify an equilibrium through trial and correction. Thus, the best strategy for game players is to imitate and improve their previous strategies. Through long-term imitation and improvement, all game players will tend to reach a definite stable strategy [31,32,33,34,35,36].

Under the mode of government supervision, there are many factors affecting the behavior of drug (food) enterprises, and many scholars have given different opinions. These research results focus on the impact of external and internal factors on their decision-making. This paper searches the fields of “drug regulation” and “food regulation” from Google academic and China HowNet, and the relevant summary is shown in Table 1:

According to the research results in recent years, it can be seen that the cost of government supervision, social benefits, benefits of drug (food) enterprises, penalties for non-cooperation, probability of public or third-party supervision are the core factors that affect the behavioral decisions of drug (food) enterprises. Therefore, based on the existing research costs, this paper took these factors as the main parameters to construct the evolutionary game model for behavioral decisions of vaccine manufacturers under different government regulatory modes.

The main problems to be solved in this paper are as follows:

(1) This paper analyzes the competition and cooperation relationship and game strategy of the behavior decision-making of vaccine enterprises under the supervision of the government, and considers the third-party supervision as an environmental factor. Combined with the analysis of variables, it discusses the conditions that affect the evolution of the interaction between vaccine manufacturers and the government towards the direction of cooperation;
(2) Based on the premise of bounded rationality, the decision-making process of vaccine manufacturing enterprises is regarded as a dynamic process of gradual learning. The evolutionary game model of government and vaccine manufacturing enterprises is constructed. The key factors affecting the game strategy of both sides are found by solving the model;
(3) By analyzing the equilibrium point and stability of the evolutionary game between the government and vaccine manufacturers, the choice of the stable strategy of the two is studied. Then, through the changes of government punishment, third-party reports, and other parameters, using MATLAB simulation analysis, the evolution trend of the game of the behavior decision-making of vaccine enterprises is investigated under different government supervision modes, and reasonable countermeasures and suggestions are put forwarded to provide decision-making basis for government departments.

## 2. Methods

### 2.1. Model Assumptions

**Hypothesis** **1: ** *Under different regulatory models, the game process between vaccine manufacturers and the government is also dynamic, and the strategies of both sides of the game can be adjusted at any time before reaching the evolutionary game equilibrium.*

**Hypothesis** **2: ** *Bribery and rent-seeking behaviors between government departments and vaccine manufacturers do not exist.*

**Hypothesis** **3: ** *In order to avoid the risk of penalties and ensure the maximization of benefits, the vaccine manufacturer will adopt the self-disciplined behavior to produce qualified vaccine with a certain probability x, so the probability of non-self-disciplined behavior is 1−x. Due to the regulatory costs of the government, the supervision of vaccine manufacturers can only take a certain probability to be carried out in active supervision strategy, and the probability of passive supervision strategy is 1−y*.

### 2.2. Model Symbol Description

In this paper, the profit and loss variables between government regulatory authorities and vaccine manufacturers are set as follows:

The government regulatory authorities need to invest certain human, material, and financial resources in the active supervision of vaccine production enterprises. This investment is affected by government power and regulations, and the resulting cost of active supervision is $\frac{C_{G1}}{\pi }$ (π is the strict coefficient of government rights and regulations). Relevant research shows that the stricter the government’s rights and regulations are, the lower the regulatory cost will be. $\frac{C_{G2}}{\psi }$ is the cost of passive supervision of government departments, which is related to the government’s corruption of government and awareness of people. Relevant research shows that the stronger the corruption of government and awareness of people, the lower the cost of passive supervision of the government. The difference between operational supervision and passive regulation lies in the fact that passive supervision refers to the supervision conducted after the report from the third party regulatory agency or the public is received. Therefore, the cost of government passive regulation is much higher than that of the active supervision cost, namely, $\frac{C_{G2}}{\psi }>\frac{C_{G1}}{\pi }>0$.

At the same time, active supervision will reduce non-self-disciplined behaviors of vaccine enterprises, which can be restrained from the source and effectively decrease social losses. Passive supervision is a type of behavior regulation after the vaccine manufacturers choose a non-self-discipline strategy, which usually causess some losses to the society. In the case of active supervision by government supervision departments, the success rate of their supervision is expressed by α (0<α≤1). In the case of passive supervision by corresponding government departments, the success rate is expressed by β (0<β≤1) and 0<α<β≤1.

Assume that λ is the probability of being reported by the third party if the enterprise is not self-disciplined. The benefits obtained by vaccine production enterprises from the self-discipline and non-self-discipline strategy are $R_{V1}$ and $R_{V2}$, respectively, where $R_{V2}>R_{V1}$. If the enterprise is not self-disciplined, the enterprise will suffer the penalty and reputation loss $F_{V1}$. Assume the penalty coefficient $\mu =\frac{F_{V1}}{R_{V2}}$ and μ>1. If the enterprise produces unqualified products and the profit is higher than the possible penalty, then the government supervision is meaningless. Assume $F_{V2}$ is the rectification cost to be paid by the vaccine manufacturer after the non-self-disciplined behavior is detected and the disposal cost to eliminate the adverse impact on the society, $C_{V}$ is the inspection cost of the vaccine manufacturers for cooperating with government supervision, and if the non-self-discipline of the vaccine manufacturer is discovered by the regulatory authorities, the government regulatory authorities will gain the benefits $R_{G}$.

The game payoff matrix of the two players is given in Table 2.

## 3. Results

### 3.1. Duplicate Dynamic Equation Construction

According to Malthusian equation, the expected benefits of vaccine manufacturers for self-discipline strategy $E_{1}^{1}$ and non-self-discipline strategy $E_{1}^{2}$, and the average benefits ${\bar{E}}_{1}$ are as follows.

(1) $$E_{1}^{1}=y(R_{V1}-C_{V})+(1-y)[\lambda (R_{V1}-C_{V})+(1-\lambda )R_{V1}]$$

(2) $$E_{1}^{2}=y[(1-\alpha )R_{V2}-\alpha (F_{V1}+F_{V2})-C_{V}]+(1-y)\{\lambda [(1-\beta )R_{V2}-\beta (F_{V1}+F_{V2})-C_{V}]+(1-\lambda )R_{V2}\}$$

(3) $${\bar{E}}_{1}=xE_{1}^{1}+(1-x)E_{1}^{2}$$

The replication dynamic equation selected by vaccine manufacturers is as follows.

(4) $$F(x)=\frac{dx}{dt}=x(E_{1}^{1}-{\bar{E}}_{1}^{})=x(1-x)\{y[(R_{V2}+F_{V1}+F_{V2})(\alpha -\beta \lambda )]+R_{V1}-R_{V2}+\beta \lambda (R_{V2}+F_{V1}+F_{V2})\}$$

Similarly, the $E_{2}^{1}$, $E_{2}^{2}$ and ${\bar{E}}_{2}^{}$ of government regulatory departments are as follows:

(5) $$E_{2}^{1}=x(-\frac{C_{G1}}{\pi })+(1-x)(\alpha R_{G}-\frac{C_{G1}}{\pi })$$

(6) $$E_{2}^{2}=x\lambda (-\frac{C_{G2}}{\psi })+(1-x)\{\lambda [\beta (R_{G}-\frac{C_{G2}}{\psi })+(1-\beta )(-\frac{C_{G2}}{\psi })]\}$$

(7) $${\bar{E}}_{2}^{}=yE_{2}^{1}+(1-y)E_{2}^{2}$$

Therefore, the replication dynamic equation of active supervision chosen by government regulatory departments is as follows.

(8) $$G(y)=\frac{dy}{dt}=y(E_{2}^{1}-{\bar{E}}_{2}^{})=y(1-y)[(\alpha -\beta \lambda )R_{G}(1-x)+\frac{\lambda C_{G2}}{\psi }-\frac{C_{G1}}{\pi }]$$

Equations (4) and (8) constitute a two-dimensional dynamic system (I).

### 3.2. Stability Analysis of Equilibrium Point

**Proposition** **1. ** *For the two-dimensional dynamic system (I), there must be $2^{2}=4$ pure strategy equilibrium points, that is (1,1), (1,0), (0,1), (0,0) respectively. At the same time, there may be an equilibrium point of a mixed strategy $\left(x^{*},y^{*}\right)$, and there is $x^{*}\in [0,1]$, $y^{*}\in [0,1]$.*

$$x^{*}=1-\frac{\lambda \pi C_{G2}-\psi C_{G1}}{\pi \psi (\beta \lambda -\alpha )R_{G}}=M,\;y^{*}=\frac{R_{V1}-R_{V2}+\beta \lambda (R_{V2}+F_{V1}+F_{V2})}{\left(R_{V2}+F_{V1}+F_{V2})(\beta \lambda -\alpha \right)}=N$$

**Proof.**  For the above two dimensional dynamical system, when x=0, x=1, y=0, or y=1, there is F(x)=0, G(y)=0. Therefore, (1,1), (1,0), (0,1), (0,0) are the equilibrium points of the system. When 0<x<1, 0<y<1, if $y[(R_{V2}+F_{V1}+F_{V2})(\alpha -\beta \lambda )]+R_{V1}-R_{V2}+\beta \lambda (R_{V2}+F_{V1}+F_{V2})=0$, and $\pi \psi (\alpha -\beta \lambda )R_{G}(1-x)+\lambda \pi C_{G2}-\psi C_{G1}=0$, then there is F(x)=0, G(y)=0. The possible equilibrium point of the two-dimensional dynamic system (I) $\left(x^{*},y^{*}\right)$ can be got by solving Equation (9), which is shown as follows. □

(9) $$\left\{ \begin{array}{l} y[(R_{V2}+F_{V1}+F_{V2})(\alpha -\beta \lambda )]+R_{V1}-R_{V2}+\beta \lambda (R_{V2}+F_{V1}+F_{V2})=0\\ \pi \psi (\alpha -\beta \lambda )R_{G}(1-x)+\lambda \pi C_{G2}-\psi C_{G1}=0 \end{array}\right.$$

According to Friedman (1998) [28], the equilibrium points can be evolutionary stable strategy (ESS for short) after the stability test, that is, the stability of the equilibrium can be judged by local stability of Jaconbian matrix.

The Jacobian matrix of the system is:

$$J=\left[\begin{array}{cc} \frac{\partial G(x)}{\partial x} & \frac{\partial G(x)}{\partial y}\\ \frac{\partial F(y)}{\partial x} & \frac{\partial F(y)}{\partial y} \end{array}\right]=\left[\begin{array}{cc} a_{11} & a_{12}\\ a_{21} & a_{22} \end{array}\right]$$

where

$a_{11}=(1-2x)\{y[(R_{V2}+F_{V1}+F_{V2})(\alpha -\beta \lambda )]+R_{V1}-R_{V2}+\beta \lambda (R_{V2}+F_{V1}+F_{V2})\}$
$a_{12}=x(1-x)(R_{V2}+F_{V1}+F_{V2})(\alpha -\beta \lambda )$
$a_{21}=y(1-y)\pi \psi (\beta \lambda -\alpha )R_{G}$
$a_{22}=(1-2y)[\pi \psi (\alpha -\beta \lambda )R_{G}(1-x)+\lambda \pi C_{G2}-\psi C_{G1}]$

If both of the following conditions are satisfied, the equilibrium point of the replicated dynamic equation is the evolutionary stability strategy (ESS).

(1) $tr\;J=a_{11}+a_{22}<0$ (trace condition);
(2) $\det\;J=\left|\begin{array}{cc} a_{11} & a_{12}\\ a_{21} & a_{22} \end{array}\right|=a_{11}a_{22}-a_{12}a_{21}>0$ (Jacobian condition)

From the above calculated data, it can be seen that $a_{11}+a_{22}=0$, and there are non-trace conditions at the local equilibrium point (M,N), so the equilibrium point is definitely not the evolutionary stability strategy (ESS) of the system. The remaining four equilibrium points are discussed below. Depending on the values of the determinant and trace of the Jacobian matrix, the local stability of the equilibrium points can be determined. The results are shown in Table 3:

**Proposition** **2. ** *Assume that $\varepsilon =\frac{R_{V2}-R_{V1}}{R_{V2}+F_{V1}+F_{V2}}$ is the ratio of profit to loss of vaccine manufacturers for their non-self-discipline strategies, where $R_{V2}-R_{V1}$ is the excess return, and $R_{V2}+F_{V1}+F_{V2}$ is the loss of vaccine manufacturers for their non-self-discipline. Therefore, from the perspective of economics, it can be regarded that ε is the ratio of profit to loss for their non-self-discipline strategies.*

**Figure 1:**
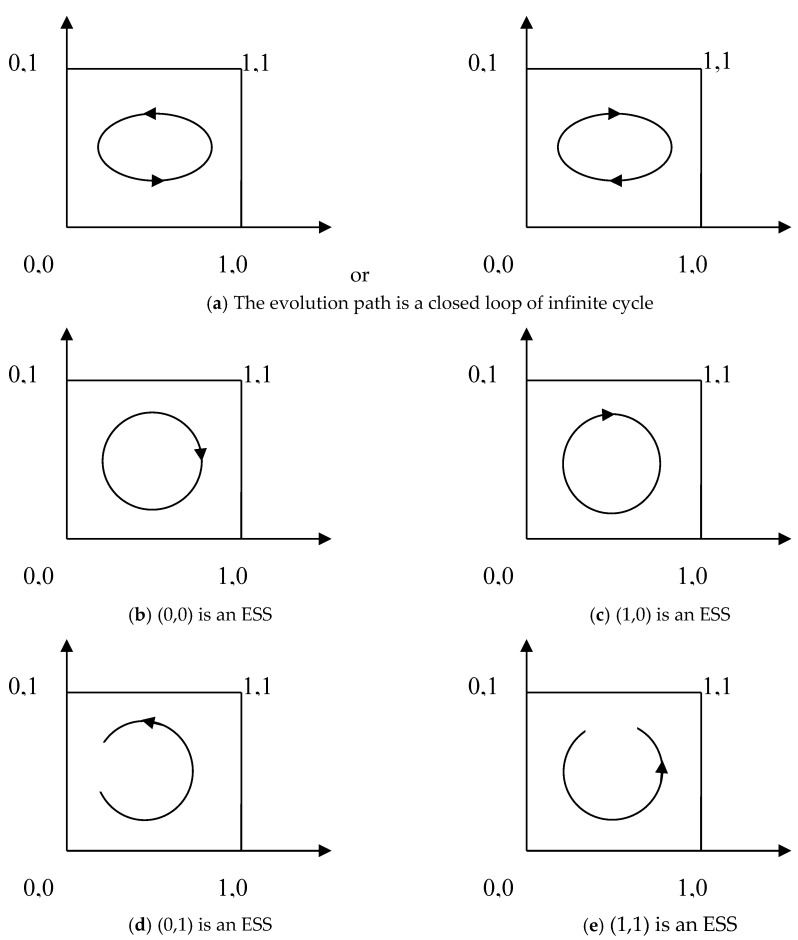
The evolution path of game strategy. (**a**) The evolution path is a closed loop of infinite cycle; (**b**) (0,0) is an ESS; (**c**) (1,0) is an ESS; (**d**) (0,1) is an ESS; (**e**) (1,1) is an ESS.

(1) When α>ε>βλ>0 or 0<α<ε<βλ, that is, when the ratio of benefit to loss of the vaccine manufacturers for their non-self-discipline is between the probabilities of success of two different regulatory approaches, it is a transition period of the game between the two sides, the two-dimensional dynamic system (I) has no ESS, and its evolutionary path is a closed circle of infinite cycles (as shown in Figure 1a).

(2) If ε>βλ>α>0 and $0<\lambda \pi C_{G2}<\psi C_{G1}$, that is, the profit loss ratio of non-self-discipline behavior strategy of vaccine manufacturers is greater than the probability of success of the two different regulatory approaches. Vaccine manufacturers will eventually choose non-self-discipline. When conducting active supervision, government regulatory departments need to invest more human resources for supervision and more financial resources to develop and purchase high-end technologies and equipment. Therefore, the cost of passive supervision is less than that of active supervision. At this point, the behavioral strategy of two players is (non-self-discipline, passive supervision), then (0,0) is an ESS of the two-dimensional dynamic system (I), and its evolutionary path is shown in Figure 1b. Similarly, when 0<ε<βλ<α and $0<\lambda \pi C_{G2}<\psi C_{G1}$, the behavioral strategy of two players is (self-discipline, passive supervision), then (1,0) is an ESS of the two-dimensional dynamic system (I), and its evolutionary path is shown in Figure 1c.

(3) If ε>α>βλ>0 and $0<\psi C_{G1}<\lambda \pi C_{G2}$, that is, the profit loss ratio of non-self-discipline behavior strategy of vaccine manufacturers is greater than the probability of success of the two different regulatory approaches. The government supervision department chooses active supervision strategy according to the principle of interests maximization, that is, the benefits received by the government regulators are greater than the gains from passive regulation. The behavioral strategy of two players is non-self-discipline and active supervision, then (0,1) is an ESS of the two-dimensional dynamic system (I), and its evolutionary path is shown in Figure 1d. Similarly, when 0<ε<α<βλ and $0<\psi C_{G1}<\lambda \pi C_{G2}$, the behavioral strategy of two players is self-discipline and active supervision, then (1,1) is an ESS of the two-dimensional dynamic system (I), and its evolutionary path is shown in Figure 1e.

It is shown that the local stability can be determined by the determinant and trace values of the Jacobian matrix of the two-dimensional dynamical system (I).

Depending on the above analysis, the two-dimensional dynamic system (I) in the case (2) and (3) in proposition 2 has corresponding evolutionary stability strategy. In particular, when α>ε>βλ>0 or 0<α<ε<βλ, the two-dimensional dynamical system (I) has no corresponding evolutionary stability strategy. According to the local stability analysis method of the Jacobian matrix, the stability analysis of its equilibrium point is carried out. The results are shown in Table 4.

Under the above assumptions, the evolutionary game model has four saddle points and a central point, which are (1,1), (1,0), (0,1), (0,0), and $\left(1-\frac{\lambda \pi C_{G2}-\psi C_{G1}}{\pi \psi (\beta \lambda -\alpha )R_{G}},\frac{R_{V1}-R_{V2}+\beta \lambda (R_{V2}+F_{V1}+F_{V2})}{\left(R_{V2}+F_{V1}+F_{V2})(\beta \lambda -\alpha \right)}\right)$.

According to the solution of the model, the characteristic roots corresponding to the point $\left(x^{*},y^{*}\right)$ are a pair of pure virtual roots. Based on the research of Taylor [42], they are stable equilibrium points of the system, but not asymptotically stable points. The evolution trajectory of the system is a closed-loop curve around the equilibrium point $\left(x^{*},y^{*}\right)$. That is to say, when α>ε>βλ>0, or when 0<α<ε<βλ, the behavioral strategy choice of vaccine manufacturers and government regulators is to keep changing around the equilibrium point $\left(x^{*},y^{*}\right)$, the system will not automatically stabilize to the equilibrium point. Therefore, the equilibrium point $\left(x^{*},y^{*}\right)$ is not the evolutionary stabilization strategy of the two-dimensional dynamic system (I).

**Proposition** **3. ** *When the profit-loss ratio of the non-self-disciplined behavior strategy of the vaccine manufacturer and the probability of success of the two different regulatory approaches meet the conditions α>ε>βλ>0 or 0<α<ε<βλ, the convergence of the system depends on the values of other parameters. According to the replication dynamic equation of the two-dimensional dynamic system (I) and the evolution phase diagram of Figure 1a, it can be seen that when the value of other parameters is fixed, the lower the proportion of self-discipline of vaccine manufacturers is, the more likely government regulatory authorities are to choose the behavioral strategy of active supervision. Similarly, the higher the proportion of active supervision by government regulatory departments, the more likely vaccine manufacturers are to choose the self-discipline strategy.*

**Proof.**  The two-dimensional dynamical system (I) of the duplicate dynamic equation is as follows. $F(x)=\frac{dx}{dt}=x(E_{1}^{1}-{\bar{E}}_{1}^{})=x(1-x)\{y[(R_{V2}+F_{V1}+F_{V2})(\alpha -\beta \lambda )]+R_{V1}-R_{V2}+\beta \lambda (R_{V2}+F_{V1}+F_{V2})\}$ $G(y)=\frac{dy}{dt}=y(E_{2}^{1}-{\bar{E}}_{2}^{})=y(1-y)[(\alpha -\beta \lambda )R_{G}(1-x)+\frac{\lambda C_{G2}}{\psi }-\frac{C_{G1}}{\pi }]$. □

It can be known that, when $y<\frac{R_{V1}-R_{V2}+\beta \lambda (R_{V2}+F_{V1}+F_{V2})}{\left(R_{V2}+F_{V1}+F_{V2})(\beta \lambda -\alpha \right)}$, there is ${\frac{dF(x)}{dx}|}_{x=1}<0$, and x=1 is the evolutionary stability strategy ESS. When $x>1-\frac{\lambda \pi C_{G2}-\psi C_{G1}}{\pi \psi (\beta \lambda -\alpha )R_{G}}$, there is ${\frac{dG(y)}{dy}|}_{x=1}<0$, and y=1 is the evolutionary stable strategy ESS. Therefore, the lower the proportion of self-discipline of vaccine manufacturers is, the higher the proportion of active supervision of government regulatory departments x will be, and vice versa.

## 4. Discussion

**Figure 2:**
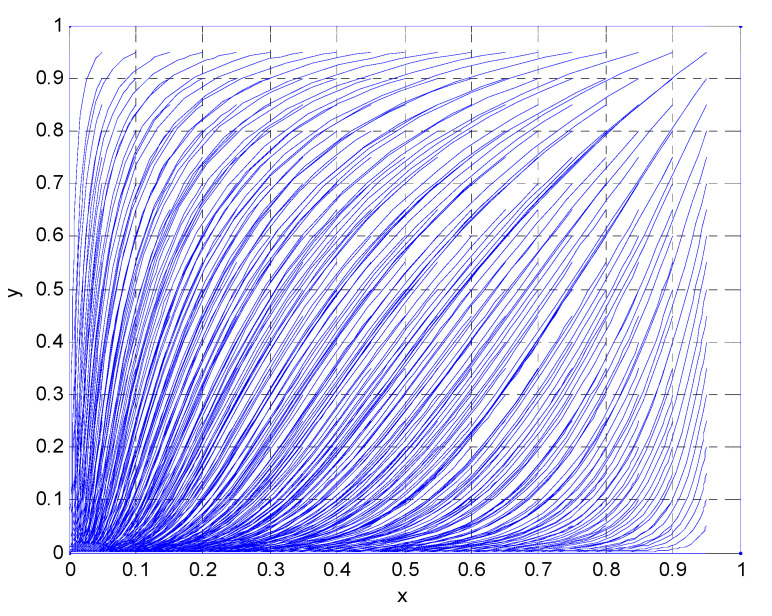
Initial state of government regulatory departments and vaccine manufacturers.

The optimal decision of the two sides is obtained through the establishment of the game model between the government regulatory department and the vaccine manufacturer. In view of the fact that although the government continuously strengthens the supervision of vaccine manufacturers, the non-self-discipline of vaccines occurs from time to time. It is of no practical significance to discuss the high incidence period of non-self-discipline and the period of enterprise self-discipline. In this context, based on the above analysis and the results, the influence of parameter changes on the strategic choice of both sides is discussed through analyzing the severity of undisciplined punishment, government active regulation, and government passive regulation. By setting relevant parameters, MATLAB R2017b software was used for the simulation to more clearly reflect the influence of parameter changes on the evolution direction of both sides. The initial value setting is shown in Table 5:

Matlab simulation was conducted based on the above parameter assumptions, and the results are shown in Figure 2. With the increase in the number of iterative steps in the simulation evolution, the evolutionary stable point of the game behavior between the government regulatory department and the vaccine manufacturer is (0,0).

### 4.1. The Impact of Government Punishment on the Evolutionary Behavior of Vaccine Companies

**Figure 3:**
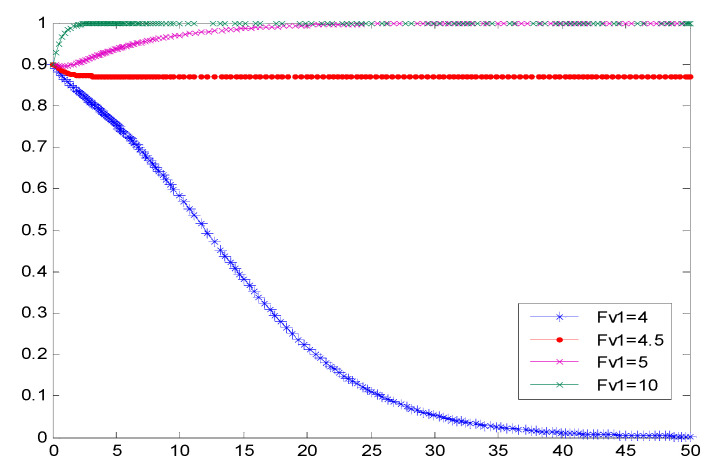
Cooperative probability simulation results of vaccine enterprises under government punishment.

According to the definition of profit–loss ratio $\varepsilon =\frac{R_{V2}-R_{V1}}{R_{V2}+F_{V1}+F_{V2}}$, it can be seen that if $\frac{\partial \varepsilon }{\partial F_{V1}}<0$, then the simulation result is inversely proportional to the simulation result ε. According to the evolution results in Figure 3, when the punishment of non-self-discipline enterprises by government departments increases continuously, the probability of enterprises choosing self-discipline will gradually increase until the self-discipline period of vaccine manufacturers is reached. According to the figure, the government regulations can effectively prevent the vaccine production enterprise non-self-discipline behavior and the government punishes the critical point $F_{V1}$ is 4.5. If $F_{V1}>4.5$, the government punishment will motivate the cooperation between vaccine enterprises; once $F_{V1}<4.5$, with the increasing extension of study time, the vaccine enterprises will eventually choose not to cooperate. That is to say, with the increase of government regulatory authorities’ punishment for undisciplined enterprises, the temptation of undisciplined behaviors ε is greatly reduced. When ε is smaller than both the probability of active regulation and the probability of passive regulation, vaccine manufacturers will enter a period of self-discipline based on their own comprehensive consideration. In the same way, the cost of the rectification after the investigation and punishment of the non-self-discipline of vaccine manufacturers can also lead to the same conclusion as that of the government punishment, which will not be repeated. When enterprises are found out to be non-self-disciplined, the government should strictly require enterprises to rectify, raise the rectification requirements, and increase the cost of rectification, so that vaccine manufacturers avoid choosing a non-self-discipline strategy.

### 4.2. The Impact of Active Regulation by Government Regulatory Departments on the Evolution of the Two Parties

**Figure 4:**
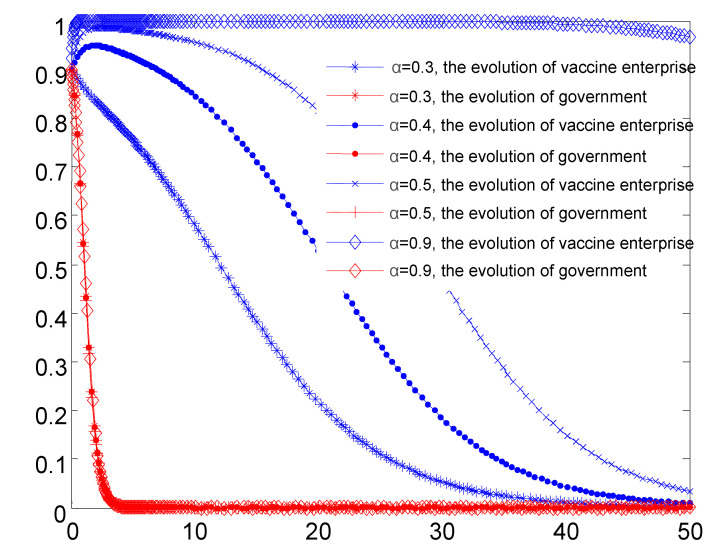
Simulation results of the evolutionary game between the two parties under the condition that the success rate of government’s active supervision is increased.

Based on the simulation shown in Figure 4, when the government regulatory active supervision rate is increasing, the vaccine production enterprise tends to choose self-discipline, and government regulators are inclined to choose passive regulation. If the government can ensure efficient regulatory success rate, the vaccine production enterprise will go constantly in the direction of self-discipline. At the same time, as the vaccine production enterprises gradually regulate their own behavior, the probability of non-self-discipline behavior appears to be smaller and smaller, and the loss to society suffering tends to be smaller as well. If the active regulation effect is not obvious, government regulators are likely to choose passive regulation with a relatively stable state, for the reason that at this time the government supervision cost and the effect of passive regulation are more favorable than that of active supervision.

### 4.3. The Influence of Passive Government Regulation and Third-Party Reporting Probability on the Evolution of Both Parties

**Figure 5:**
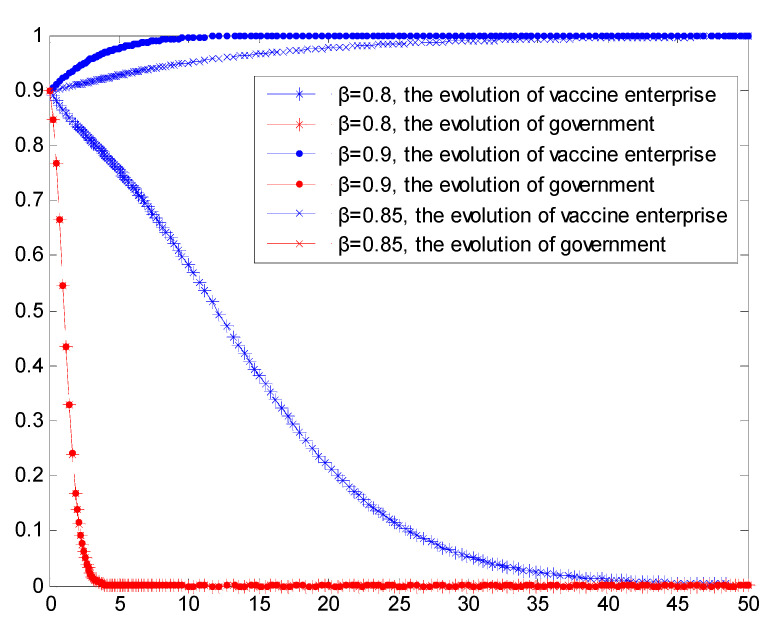
Simulation results of the evolutionary game between the two parties under the circumstance that the success rate of government passive supervision is increased.

**Figure 6:**
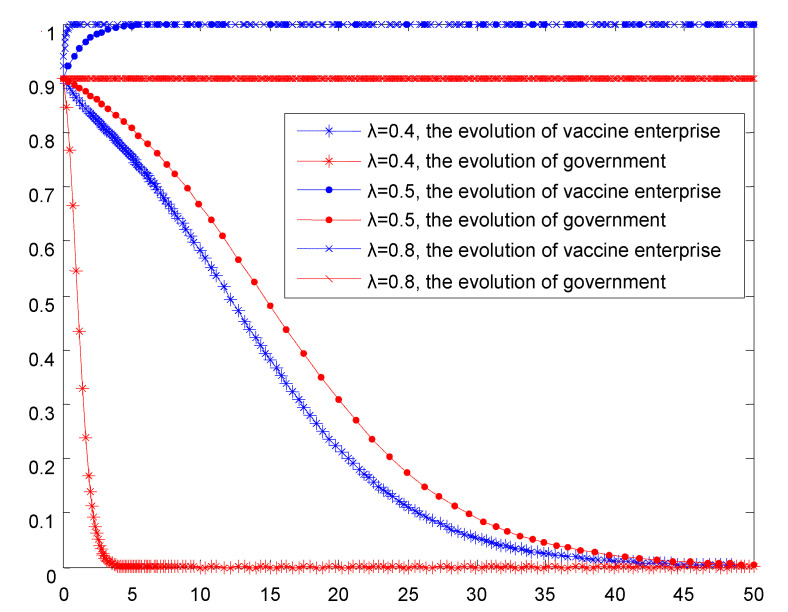
Simulation results of the evolutionary game between the two parties under the condition that the probability of third-party reporting is improved.

Depending on the simulation in Figure 5 and Figure 6, with the increase of the probability of passive regulation and reports from the third party, the non-self-disciplined behavior of vaccine production enterprises is more likely to be exposed, which is a great threat to the enterprise. In the case of severe punishment by the government regulatory authorities, the possibility of investigation and punishment faced by enterprises is also increasing, which will make enterprises face high cost of punishment, and ultimately vaccine manufacturers choose self-discipline behavior. With the increase in the success rate of passive regulation and the report of the third party, governments tend to adopt a passive regulation strategy. In line with the principle of the minimum loss to society, the government supervision department should take the initiative and increase the success rate of functional regulation to minimize non-self-discipline choices of the vaccine production enterprises and avoid damage to the society.

### 4.4. The Influence of Power of the Government and Regulation on the Evolution of Both Parties

**Figure 7:**
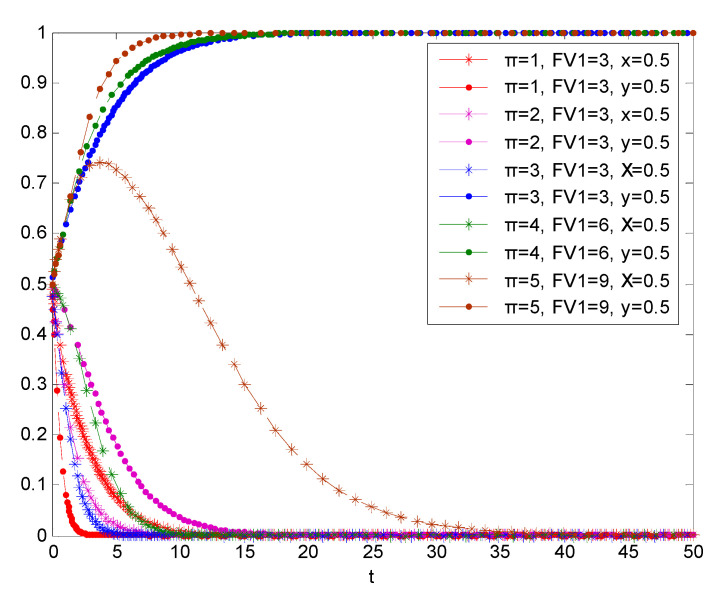
Simulation results of the evolutionary game between the two parties under the condition that power of the government and regulation is improved.

Based on the simulation in Figure 7, when the power of government is greater and the regulations are stricter, the regulatory cost of the government’s regulatory authorities will be reduced due to the deterrence of vaccine manufacturers to the government’s power and regulations. At this time, the government will choose to take the initiative due to the lower cost of active regulation. At this time, due to strict government power, laws and regulations, vaccine enterprises will increase the penalty and reputation loss when they do not self-discipline. At this time, in order to avoid greater loss, vaccine enterprises will take the principle of maximizing their own interests, and finally turn from non-self-discipline behavior strategy selection to self-discipline behavior. Therefore, greater government power and strict laws and regulations are conducive to promoting the active supervision of the government regulatory authorities and the cooperative behavior of vaccine enterprises.

### 4.5. The Influence of Corruption of Government and Awareness of People on the Evolution of Both Parties

**Figure 8:**
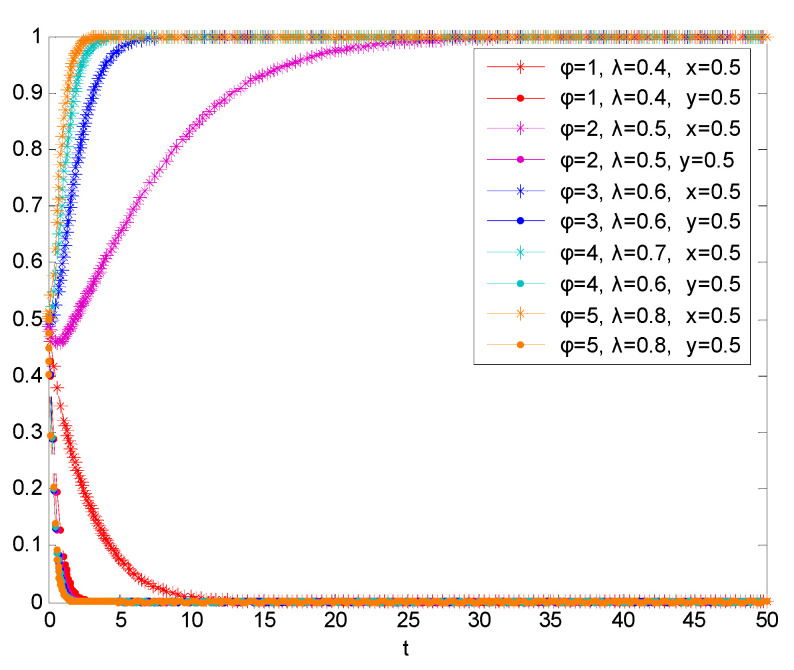
Simulation results of the evolutionary game between the two parties under the condition that corruption of government and awareness of people is improved.

Based on the simulation in Figure 8, the larger the coefficient of corruption of government and awareness of people, the lower the cost of passive supervision of the government regulatory department, and it will also tend to avoid the cost and choose passive supervision. Because the opportunity cost of the government’s active supervision is higher, the final choice is passive supervision. However, due to the high level of corruption of government and awareness of people, vaccine enterprises will increase the probability of third-party reporting, which will increase the cost of non-self-discipline, and finally choose self-discipline strategy. Therefore, corruption of government and awareness of people are also conducive to promoting the passive supervision of government regulatory departments and the cooperative behavior of vaccine enterprises.

## 5. Conclusions

According to the above analysis, the main conclusions can be drawn: (1) Severe punishment by government regulatory authorities, and increased costs of rectification after investigation and handling of involuntary behaviors of vaccine enterprises can effectively prevent involuntary behaviors of vaccine enterprises. (2) In the early stage of the game, the success rate of the government’s efficient supervision will make the vaccine enterprises continuously self-disciplined; when the vaccine market is relatively stable, the government’s supervision departments tend to be more conducive to passive supervision. (3) The success rate of government regulatory departments and the probability of third-party reporting play a great role in promoting the self-discipline of enterprises. Once the phenomenon of vaccine enterprises’ non-discipline increases, the government regulation must change from passive regulation to active regulation. (4) The power of government and regulation are conducive to promoting the active supervision of the government regulatory authorities, but corruption of government and awareness of people are different.

Built on the above conclusions, the following policy suggestions are proposed for the government to guide and supervise the vaccine manufacturers to take self-discipline actions.

### 5.1. Raising Public Awareness of Public Safety

Make full use of the publicity advantages of new media platforms, strengthen the publicity and education of vaccine safety knowledge, and improve public safety awareness. Specifically, continuously strengthen the team construction of the vaccine regulators and use new media software through various channels to improve the public’s ability to identify fake and substandard vaccines. Strengthen public awareness and safeguard their legitimate rights and interests of consumers to report complaints of their own, so as to establish the corresponding reporting incentives and actively guide the public to participate in supervision. Encourage and support the vaccine production line staff in their disclosure regarding vaccine quality and safety problems in a timely manner.

### 5.2. Severe Punishment

Heavy penalties for non-self-disciplined vaccine production enterprises should be enforced according to specific situations. For a large-scale vaccine production enterprise, two hundred million RMB is not significant, while it is too much to bear for a small-scale vaccine production enterprise. Therefore, one of the principles that must be adhered to is to hit the “pain point” of the non-self-discipline vaccine manufacturers. Severe punishment will be meted out to the vaccine manufacturers that refuse to change.

### 5.3. Improving Government Supervision

Government supervision includes active supervision and passive supervision. Active supervision requires the government to continuously increase capital investment in vaccine safety supervision, and urges food and drug safety supervision departments in various regions to conduct regular and irregular inspections on local vaccine manufacturers with high frequency and intensity. Passive supervision is mainly third-party supervision agencies and public supervision. The involvement of third-party regulatory agencies is a change from the traditional government regulatory model. With the introduction of a third-party regulatory agency, vaccine regulation is linked with “Internet plus”. Through the use of various information technologies, the vaccine safety supervision information platform can monitor vaccine manufacturers in real time, increase the depth and breadth of supervision, and realize the traceability of vaccines in various circulation links, which can effectively improve the supervision efficiency. Public supervision is an important part of vaccine safety supervision. When the government is in a state of passive supervision for various reasons, public supervision can effectively improve the success rate of the government’s passive supervision. Although the cost of government’s passive supervision is higher than that of active supervision, timely loss control is another way of self-protection. Public supervision is an effective supplement to government supervision. Multiple forms of supervision coexist to achieve multiple co-governances.

In this paper, we investigate the evolutionary game of behavior decision-making of vaccine enterprises under the supervision of the government, and analyze the interaction of behavior between the players. However, we have focused on the situation where there are only two strategies for the players. In future research, we will consider the evolutionary game involving multi players and multi strategies, and the impact of government policies and strategies on the behavior decision-making of heterogeneous vaccine enterprises.

## Figures and Tables

**Table 1:** Influencing factors of drug (food) enterprise behavior decision under government supervision mode.

Scholars (Year)	Influencing Factors
Song Yan (2009) [37]	Income from qualified drugs and fake drugs.
Cao Jiantao (2018) [13]	Additional benefit, benefit of inaction, positive utility, cost of implementation, government punishment.
Song Yan (2016) [15]	Benefits of qualified drugs, safety costs, benefits of fake and inferior drugs, accident handling costs, government fines.
Fang Yu (2010) [16]	Regulatory probability, regulatory cost, safety accident probability, violation cost, disposal cost, discount factor, social loss, penalty amount.
Yan Jianzhou (2015) [18]	Regulatory cost, safety cost, penalty, legal liability cost, social cost, reputation loss.
Liu Sukun (2011) [38]	Regulatory costs, normal earnings, excess earnings, penalties.
Liu Sukun (2012) [39]	Supervision cost, reputation loss, degree of supervision, illegal earnings. Punishment, enterprise earnings, probability of illegal operation, probability of legal operation, probability of public report and verification of earnings.
Jiang Shubo (2009) [40]	Control cost, penalty amount, compensation amount, regulatory probability. Disguised cost, income from superior goods, income from inferior goods, excess income.
Zhao, L (2018) [41]	Government regulations, product output, product revenue, regulatory cost.

**Table 2:** Game payoff matrix.

Game Players		Government Regulatory Authority	
		Active Regulation(y)	Passive Regulation(1−y)
vaccine manufacturers	Self-discipline (x)	$R_{V1}-C_{V}$, $-\frac{C_{G1}}{\pi }$	$\lambda (R_{V1}-C_{V})+(1-\lambda )R_{V1}$, $\lambda (-\frac{C_{G2}}{\psi })$
	non-self-discipline (1−x)	$(1-\alpha )R_{V2}-\alpha (F_{V1}+F_{V2})-C_{V}$, $\alpha R_{G}-\frac{C_{G1}}{\pi }$	$\lambda [(1-\beta )R_{V2}-\beta (F_{V1}+F_{V2})-C_{V}]+(1-\lambda )R_{V2}$, $\lambda [\beta (R_{G}-\frac{C_{G2}}{\psi })+(1-\beta )(-\frac{C_{G2}}{\psi })]$

**Table 3:** Determinant values and trace of local equilibrium points.

Local Equilibrium Points	$a_{11}$	$a_{12}$	$a_{21}$	$a_{22}$
(0,0)	$R_{V1}-R_{V2}+\beta \lambda (R_{V2}+F_{V1}+F_{V2})$	0	0	$\pi \psi (\alpha -\beta \lambda )R_{G}+\lambda \pi C_{G2}-\psi C_{G1}$
(0,1)	$R_{V1}-R_{V2}+\alpha (R_{V2}+F_{V1}+F_{V2})$	0	0	$-[(\alpha -\beta \lambda )R_{G}+\lambda \pi C_{G2}-\psi C_{G1}]$
(1,0)	$-[R_{V1}-R_{V2}+\beta \lambda (R_{V2}+F_{V1}+F_{V2})]$	0	0	$\lambda \pi C_{G2}-\psi C_{G1}$
(1,1)	$-[R_{V1}-R_{V2}+\alpha (R_{V2}+F_{V1}+F_{V2})]$	0	0	$-(\lambda \pi C_{G2}-\psi C_{G1})$
$\left(x^{*},y^{*}\right)$	0	*M*	*N*	0

**Table 4:** Stability analysis equilibrium points when α>ε>βλ>0 or 0<α<ε<βλ.

Local Equilibrium Points	det J	tr J	Results
(0,0)	-	uncertain	Saddle point
(0,1)	-	uncertain	Saddle point
(1,0)	-	uncertain	Saddle point
(1,1)	-	uncertain	Saddle point
$\left(x^{*},y^{*}\right)$	+	0	Center point

**Table 5:** Parameter initial value setting.

Parameter	$C_{G}{}_{1}$	$C_{G}{}_{2}$	$R_{G}$	α	β	λ	$R_{V1}$	$R_{V}{}_{2}$	$F_{V1}$	$F_{V2}$	$C_{V}$	π	ψ
**Value**	1	5	3	0.3	0.8	0.4	2	6	4	2	1	1	1
